# The Weight of Lead: Effects Add Up In Adults

**DOI:** 10.1289/ehp.115-a30

**Published:** 2007-01

**Authors:** Angela Spivey

Lead toxicity is not a problem of the past, nor is it the exclusive domain of children. In fact, lead continues today to pose a serious threat to the health of many U.S. adults.

It’s true that in the United States, environmental lead levels are much lower than before the toxic metal was removed from gasoline, food cans, and other products in the 1970s and early 1980s. The National Health and Nutrition Examination Surveys have shown that average adult blood lead levels have declined from about 15 μg/dL in the 1970s to today’s 1–2 μg/dL. But there are still pockets of high exposures, such as among workers in certain industries.

Despite reductions in exposure following OSHA’s 1978 publication of lead standards for general industry, more than 80% of elevated lead levels in adults come from workplace exposures. Industries most affected include lead mining, refining, and smelting; construction work involving paint removal, demolition, and maintenance of outdoor metal structures such as bridges and water towers; auto repair; and battery manufacturing and recycling.

When workplaces adhere to the OSHA standard, occupational exposures are usually reduced below levels that cause symptomatic lead poisoning. But as far back as 1990, studies have suggested that significant health effects happen at levels below those allowed by OSHA. “Historically people had huge lead exposures, so the OSHA standard, when it was originally established, was protective. But right now nobody thinks that that’s a protective standard,” says Rosemary Sokas, director of the Division of Environmental and Occupational Health Sciences at the University of Illinois at Chicago School of Public Health.

Now scientists say the evidence is overwhelming that action needs to be taken to further reduce lead exposures in both the workplace and the general environment. “What’s driving concern over the need to reduce permissible levels of exposure in the workplace are . . . more subtle or chronic problems such as hypertension, and contributions to cognitive dysfunction,” says Michael Kosnett, an associate clinical professor of clinical pharmacology and toxicology at the University of Colorado Health Sciences Center.

With the lower levels of lead found in the general population in the United States, much of the worry is about lead’s health effects over the long haul. The most recent evidence from epidemiological and toxicological studies suggests that low levels of exposure can, over time, damage the heart, kidneys, and brain. Some of these health effects, such as a 1-mm rise in blood pressure or a slight cognitive decline, seem small when expressed as the average impact to the entire population. In one individual, they may not even be noticed. But the overall impact on public health nevertheless worries scientists.

## Better Tools to Measure Smaller Effects

Tests to measure lead exposure itself and its health effects have become more sophisticated. The blood lead level, long the gold standard for assessing risk, reflects the amount of lead circulating in the body at the time of the test but may not offer a reliable indication of an individual’s past or cumulative exposure. For example, similar blood lead concentrations in two individuals (or populations) do not necessarily translate to similar exposure histories. One reason is that the body stores lead in the bone, and it’s released from the bone into the blood at differing rates, depending on age, gender, and other factors. For instance, lead will mobilize from bone more quickly in people with conditions in which the body is resorbing bone, such as pregnancy or osteoporosis.

Stores of lead in bone are a more reliable marker of cumulative lead exposure. In the late 1980s a noninvasive way of measuring bone lead emerged, using X-ray fluorescence technology. Scientists began applying the technique in epidemiological studies in the 1990s. But since the technology is available at only a handful of institutions in the United States, it isn’t currently feasible for routine medical management.

Measurement of lead’s health effects have improved as well. Studies of cognitive function, for example, now have the benefit of more sensitive markers, including tests of memory, visuospatial function, and the ability to communicate or understand communication. In addition, studies with larger sample sizes and those that look at community-based populations, not just occupationally exposed workers, have sharpened the picture of the effects. “Industry studies of workers suffer from a number of methodological limitations, such as the inability to follow workers who leave the industry,” says Howard Hu, chairman of the Department of Environmental Health Sciences at the University of Michigan School of Public Health.

Much of the evidence in humans comes from epidemiological studies, which show associations between lead and health effects, although alone they don’t definitively prove causation. But animal studies support many of these findings and suggest mechanisms for some of these health effects.

## Putting Pressure on the Heart

According to Stephen Rothenberg, a senior researcher at Centro de Investigación y de Estudios Avanzados-Mérida in Yucatán, Mexico, the cardiovascular system is the most thoroughly studied system in adults in terms of lead’s effects. A large number of these studies have investigated the effects of lead on blood pressure. Increases in both blood lead and bone lead appear to be associated with blood pressure increases.

Many epidemiological studies in humans suggest that rising blood lead correlates with rising blood pressure. Overall, most epidemiological studies of the general population have shown a 1-mm increase in systolic pressure for every doubling of blood lead, and this increase has been seen at a range of concentrations, from 1 to 40 μg/dL. “When applied to large numbers of people,” Rothenberg says, “those increments shift the blood pressure curve to the right, to higher values, meaning that anywhere from tens or hundreds of thousands more people . . . to tens to hundreds of millions more are going to have blood pressures that are higher than most physicians currently think is safe.”

In the last 15 years, about a dozen studies have also tied bone lead to blood pressure increases. For example, in a longitudinal study published in the 15 January 2001 *American Journal of Epidemiology*, Yawen Cheng of Harvard Medical School and colleagues found that in men who began the study without hypertension, baseline bone lead level predicted development of the condition six years later.

Rothenberg points out that although blood lead primarily reflects recent exposures, it can also in part reflect the leaching of bone lead stores back into the bloodstream. “The combination of these two results—significant blood lead effects on blood pressure, and significant bone lead effects on blood pressure—makes researchers feel that past exposures, especially when current exposure is low, may be the dominant factor in determining lead effects on blood pressure,” he says.

Lead is also associated with increased mortality from diseases of the heart. In a study published 26 September 2006 in *Circulation*, Andy Menke of Tulane University and colleagues found an increased risk of death from all causes as well as from cardiovascular disease and stroke in association with blood lead concentrations as low as 2 μg/dL. The study analyzed data from more than 13,000 participants in the Third National Health and Nutrition Examination Survey Mortality Study.

An editorial accompanying the *Circulation* article points out some of its limitations, including the fact that participants’ mortality could have resulted in part from higher lead exposures that occurred prior to the study period. But the editorial also states that the report “breaks new ground by extending the dose–effect relation to considerably lower blood lead concentrations than reported in previous studies.”

Study coauthor Paul Muntner, an associate professor of epidemiology at Tulane University, says these associations stayed “remarkably consistent” across subgroups, including smokers, diabetics, males, and females. The consistent results suggest the associations aren’t likely due to chance or other artifacts, he says.

So, how does lead actually cause cardiovascular effects? Animal studies show that lead can promote the growth of vascular smooth cells, which play a role in the formation of atherosclerotic plaques. Lead’s promotion of oxidative stress is thought to play a role in its cardiovascular effects (as it is in other lead-linked health effects). Oxidative stress happens when chemically reactive oxygen and nitrogen damage cells in a process similar to how oxygen rusts metal.

Damage from lead-induced oxidative stress has been demonstrated in studies of rats as well as in studies of human cells in culture, says N.D. Vaziri, chief of the Division of Nephrology and Hypertension at the University of California, Irvine, Medical Center. In Vaziri’s studies of human endothelial cells, for instance, development of oxidative stress has been seen immediately after lead exposure.

But in animals it takes 10 to 12 weeks for lead exposure to result in hypertension, and in humans it likely takes years to decades, Vaziri says. One possible reason is that the body launches a variety of defense mechanisms that prevent or minimize rapid rise in blood pressure and gross tissue damage. However, over time, these defense mechanisms gradually fail, blood pressure begins to rise, and detectable tissue damage appears.

Vaziri demonstrated this progression in cell culture studies published in the May 2000 issue of the *American Journal of Hypertension*. Immediately after lead exposure, the levels of a free radical called superoxide rose, “but then the cells are able to defend themselves,” Vaziri says. “A day later, the superoxide went down, but an enzyme that is made to capture the free radicals and temporarily prevent them from causing damage—the enzyme called superoxide dismutase—went up. So we had a reduction in superoxide at an interim period. But the defending enzyme converts superoxide to hydrogen peroxide, which is less toxic than the original free radical but still toxic, and capable of causing injury and dysfunction upon prolonged exposure.” Thus, Vaziri explains, the organism mounts a defense that is able to, at least for the time being, prevent the expression of disease and injury as such. Ultimately, however, lead exhausts the system.

## Filtering the Evidence on Kidney Function

Epidemiological studies of the general population suggest that kidney function may be altered at the lowest levels of blood lead studied to date in relation to renal effects. In a review published in the December (2) 2006 issue of *Kidney International*, E.B. Ekong and colleagues at The Johns Hopkins University wrote that lead contributed to kidney damage at concentrations below 5 μg/dL. “Many different studies—in Europe, Asia, and the United States—have shown that higher blood lead levels are associated with lower creatinine clearance, indicating worse kidney function,” says Virginia Weaver**,** an associate professor of environmental health sciences at Johns Hopkins and one of the authors of the review.

Some of these studies have been longitudinal, which helps address a chicken-and-egg question: is kidney damage associated with higher blood lead levels because lead causes the damage, or because damaged kidneys can’t excrete lead? “If you look at the longitudinal data, initial lead level predicts subsequent decline in renal function,” Weaver says.

In addition, lead’s effects on the kidneys are thought to play a major role in its effect on blood pressure. This is because the kidneys help regulate blood volume and vascular tone, which are the principal determinants of blood pressure. “The kidney is the pathway through which we get rid of the excess salt and fluids. Consequently, impairment of the kidney’s ability to efficiently excrete salt and fluids can result in the rise in blood volume and, hence, blood pressure,” Vaziri says. “Also, the kidney produces hormones that regulate the tone of blood vessels. Thus, alterations of kidney function or structure can cause the blood vessels to constrict throughout the body, thereby raising blood pressure.”

## Small But Measurable Cognitive Declines

Some studies of lead workers have shown associations between blood lead concentrations of 20 to 40 μg/dL and subclinical cognitive decline, including changes in memory or mental processing speed that are measurable but don’t put an individual outside the normal range of function. “Effects of lead at these levels may be such that in any one person they wouldn’t be able to notice a difference,” says Kosnett. But as with cardiovascular effects, when averaged across the whole population, there’s a measurable effect.

Declines in cognitive function are more likely to be associated with lower-level environmental exposures over time, rather than recent acute exposures. “Some of the literature suggests that lead may contribute to or accelerate an age-related decline in cognitive function,” Kosnett says. “That may be a consequence of cumulative lead exposure.”

One subgroup especially vulnerable to the effects of low-level lead exposure are pregnant women, whose exposure may affect their offspring’s cognitive function. In the May 2006 issue of *EHP*, Lourdes Schnaas of the Mexican National Institute of Perinatology and colleagues published one of the few studies of this relationship to pinpoint prenatal lead exposure as a greater risk to offspring IQ than childhood exposure. Previous studies had shown the strongest effects with postnatal exposure. In the *EHP* study, though, prenatal exposure had a more significant effect than postnatal exposure, and the strongest effects were seen at the lowest levels of exposure, says Rothenberg, a coauthor on the study. The pregnant women’s blood lead concentrations ranged from 1 to 33 μg/dL, with a mean level of 8 μg/dL.

In other neurotoxic effects, animal studies have suggested that lead exposure increases the risk of brain cancer. Some association studies in humans also have suggested a link. For instance, a 1 September 2006 report in the *International Journal of Cancer* by Edwin van Wijngaarden, an assistant professor of community and preventive medicine at the University of Rochester, showed that workers in jobs with high lead exposure were more likely than unexposed subjects to die from brain cancer. The study’s value lies in its large sample size; it analyzed the lead–brain cancer death association among more than 300,000 subjects in the National Longitudinal Mortality Study, a prospective census-based study of the U.S. population. But the study did not measure actual lead exposure; instead, Wijngaarden used participants’ self-reported occupations and the job exposure matrix developed by the National Cancer Institute (NCI) to estimate lead exposure. “This data set and the occupational job exposure matrix that NCI has come up with certainly gave me a lot more statistical power than any of the studies [regarding lead exposure and brain cancer] that have been published so far,” Wijngaarden says.

Wijngaarden is now recruiting participants for a pilot study in which he will measure bone lead in patients with brain tumors. “The main goal is to get a system ready for a larger study and to get preliminary data,” he says. No studies to date have measured bone lead in cancer patients.

Despite these findings, however, studies of brain cancer and lead have been inconsistent—some studies have found elevated rates of brain cancer associated with lead exposure, and some have not. For cancer in general, most studies show a positive association between low levels of lead exposure and cancer, but with relatively few cancer deaths, says Kyle Steenland, a professor of environmental and occupational health at Emory University.

## Is There Any Safe Level of Lead?

Current research hasn’t been able to determine a threshold for many of lead’s effects. That is, scientists haven’t yet found a concentration of lead below which no effect occurs. Some scientists say that determining a threshold would require long-term prospective studies of adults with blood lead levels commonly found in the current population. “We need to be able to characterize the dose–response curve at very low levels of exposure if these data are going to be used to intelligently plan regulation,” Rothenberg says.

Scientists disagree on just how low measurements need to go. “Is it going to do us much good to reduce the standard for intervention for kids or pregnant women from ten to five [μg/dL], or do we really have to get down to one or below in order to prevent measurable damage?” says Rothenberg. Further, policy makers will want to know if there is a level of blood lead below which the resulting improvement in public health no longer outweighs the cost of further exposure reduction. “We won’t know where that turnover point in the cost–benefit function is until we include studies that reliably measure blood lead in many subjects below point-one micrograms per deciliter,” he says.

Rothenberg suggests that scientists should take advantage of advanced technologies such as inductively coupled plasma–mass spectrometry to measure ultralow levels of blood lead—below 1 μg/dL. That technology isn’t widely available, and it’s at least five times more expensive than current methods used to measure blood lead.

Hu agrees that to better define risk, prospective studies are needed of adults with low to modest lead exposure (in the blood lead range of 1 to 10 μg/dL). But he believes that studying levels below 1 μg/dL would be “overkill.”

Weaver cautions that though population studies clearly show health effects at blood lead levels below 5 μg/dL, it’s hard to rule out the possibility that those health effects were caused by past higher exposures. “We don’t know if this is a cohort effect and how much of these health effects will further decrease as lead exposure continues to decline,” she says.

At least one longitudinal study supports the idea that health effects seen at low blood lead levels aren’t artifacts of higher past exposures. The Normative Aging Study, conducted among more than 2,000 men in Boston**,** showed lasting renal effects in a group that was known to have maintained blood lead levels below 10 μg/dL since 1979. “They found some of the strongest associations in that group,” Weaver says. Still, the group could have had higher exposures before 1979.

“That’s always the trick with a cumulative toxicant,” Weaver says. “The blood levels you see today could have been much higher in the past. You don’t have any way of telling unless you do bone lead measurements in everyone.”

## Medical Management

Physicians can get some idea of cumulative exposure by measuring blood lead regularly. “For any employer or employee who is facing a job in which there’s lead exposure, taking regular blood leads, keeping those records, and periodically calculating a cumulative exposure index is inexpensive and reliable,” Hu says.

What’s a reasonable blood lead level in someone who’s exposed to lead on the job? Hu says that at levels at or below 20 μg/dL, a worker is assuming some increased risk but an amount that may be acceptable. “If you have a blood lead of twenty for a working lifetime—let’s say forty-five years—you will get a cumulative blood lead index that we calculated would be equivalent to a bone lead of a certain level,” Hu says. “That bone lead level in our epidemiology studies still corresponds to a certain excessive risk of developing hypertension and declined cognition. But it’s not greatly above what the general population sees.”

Kosnett recommends that at occupational exposures as low as 10 μg/dL, physicians should increase monitoring and reduce lead exposures. He recommends removing people from all lead exposure when blood lead levels are at 20 μg/dL and remain there when a second measurement is taken four weeks later, or if a single check registers a level of more than 30 μg/dL.

By contrast, OSHA’s current lead standard doesn’t require full removal from exposure until blood lead concentrations exceed an average of 50 μg/dL over three successive tests or two back-to-back measurements of 60 μg/dL. Scientists have been calling for reductions in these cutoffs as far back as 1991. But OSHA has long been reluctant to revise standards proactively, says Sokas, who once served as chief medical officer at OSHA.

“The lead standard is very strong in terms of wage replacement if blood lead is above a certain cut line,” says Kenneth Rosenman, chief of the Division of Occupational and Environmental Medicine at Michigan State University. And the standard does include a provision that, even at blood lead levels below the mandatory cutoffs, physicians can recommend medical removal when workers have a specific medical condition, with these workers entitled to the same job and salary protection as those whose blood lead levels rise above the 50/60 μg/dL cutoff.

Still, although that provision protects against overt lead poisoning, it does nothing to promote preventative removal at lower levels—such as 20 μg/dL—that may pose long-term health risks. By the same token, there’s nothing in the law that prohibits a pregnant woman from working with lead until her blood level reaches 60 μg/dL. Indeed, a 1991 Supreme Court decision in the case of *Automobile Workers v. Johnson Controls* held that precluding such women from exposure would be unlawfully discriminatory. “So on the books now, it’s still acceptable for a pregnant woman to be highly exposed to lead to the same degree as men, even though the evidence is overwhelming that fetuses are exquisitely vulnerable to lead,” Hu says.

In the past, OSHA has touted its emphasis on voluntary medical surveillance programs and other measures to reduce exposure. Industry tends to favor voluntary exposure reduction as well. “The science has developed since the last time the OSHA standard was visited, and we’re aware of that,” says David Weinberg, counsel for the Battery Council International. “I’m not sure it’s necessary that OSHA reopen the lead standard. The battery industry and the secondary smelter industry have worked pretty hard on these issues, and have worked with and expect to continue to work with OSHA and others in voluntary programs to make sure that the progress that’s been made continues.”

## Protection for the Most Vulnerable

Other subgroups that are very susceptible to lead exposure are emerging, and scientists say these groups pose another reason that regulations should be strengthened. People with certain genetic susceptibilities might constitute one such group. “It’s been recognized for a long time that there can be considerable interindividual variability in people’s susceptibility to the development of symptomatic lead poisoning,” Kosnett says.

Even at a blood lead level as high as 60 μg/dL, for instance, some people will show symptoms and others won’t. Now, scientists are finding that the same is true of the emergence of health effects at very low levels of lead exposure, and recent research suggests that genetic variations may play a role. **“**Further research is needed to explore and understand this aspect of gene–environment interaction,” Kosnett says.

People who already have medical conditions are also at increased risk. In the November 2006 issue of *EHP*, Hu and colleagues showed that lead exposure was associated with increased heart rate variability (an indicator of poor cardiovascular health), especially in people with metabolic syndrome, a cluster of conditions including obesity, high blood sugar, and high blood pressure. People with any or all of these conditions are also known to be at increased risk for kidney damage and so may be more susceptible to lead’s effects. “With the obesity epidemic in so many countries, diabetes and hypertension are increasing, so we do have more groups at risk,” Weaver says.

Such studies point to another reason why lead exposure is very much a problem of the present. “As a nation, the work force is aging, and we’re expecting ourselves and our workers to keep working when they’re older,” Hu says. “But that means that a lot of them will have medical conditions, and we have to anticipate their vulnerability to environmental risk factors like lead.”

Muntner says that more work is needed to find effective and safe interventions for lowering lead exposure at the population level for people whose blood lead concentrations are already below 10 μg/dL. He points out that although blood lead levels have decreased substantially in the last 30 years, they are still much higher than they were in preindustrial times, before humans began spreading lead into the air, water, and soil.

“So we need to not be complacent and say, ‘We’ve lowered lead,’ but rather we need to think about it in terms of how can we reduce lead more, such that we eliminate this environmental toxicant,” Muntner says. “There’s really no biological function of lead, and there’s really no reason why we should be exposed to it.”

## Figures and Tables

**Figure:**
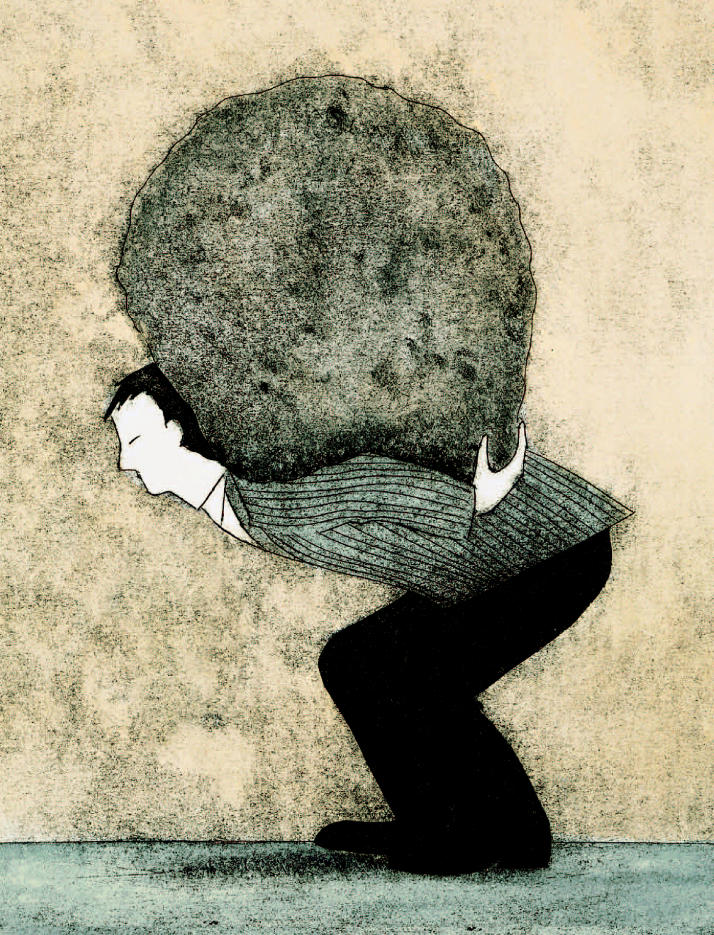


**Figure:**
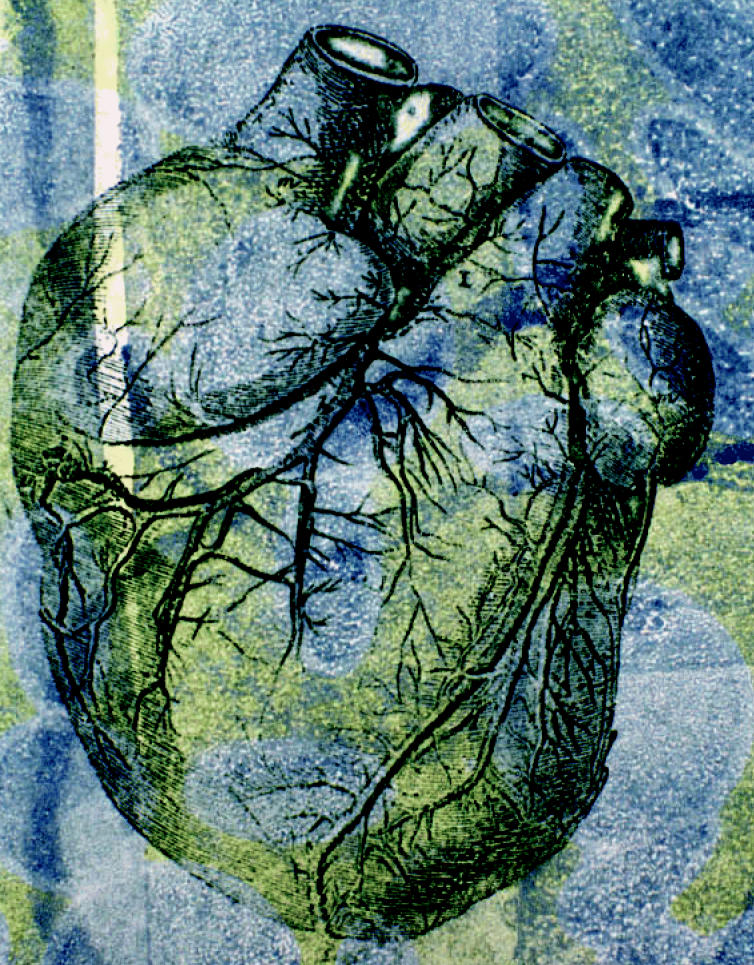
Pressure point. Increases in both bone lead and blood lead appear to be associated with possibly dangerous increases in blood pressure.

**Figure:**
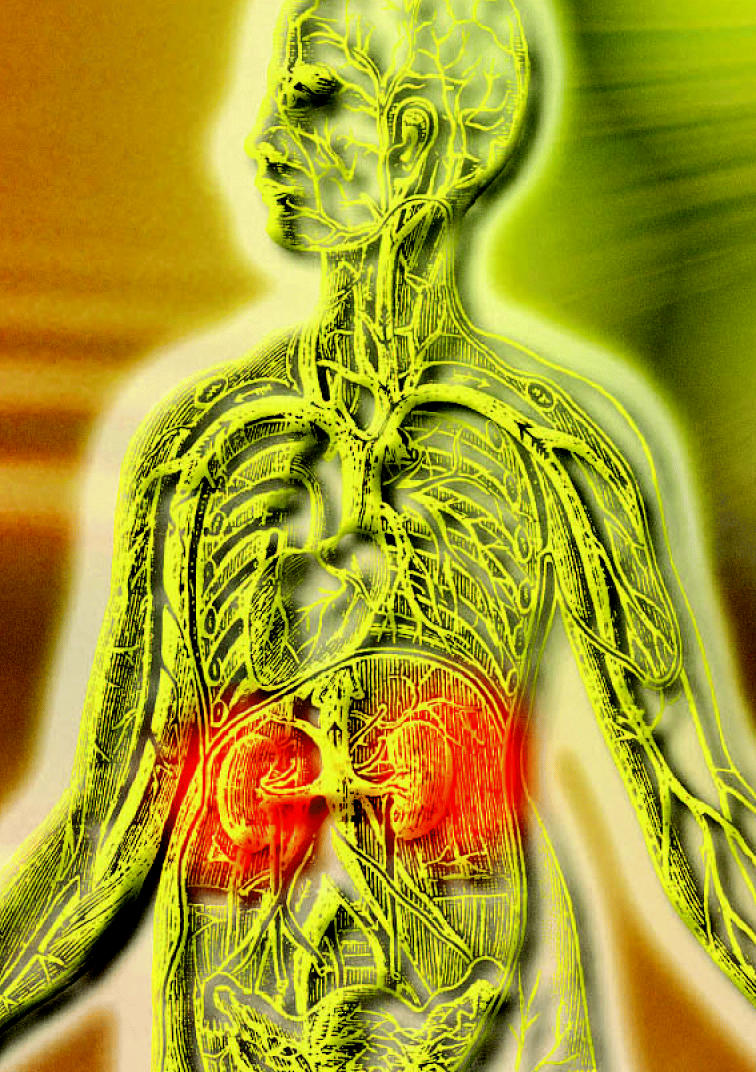
Renal reality. Studies show that higher blood lead concentrations are linked to decreases in kidney function.

**Figure:**
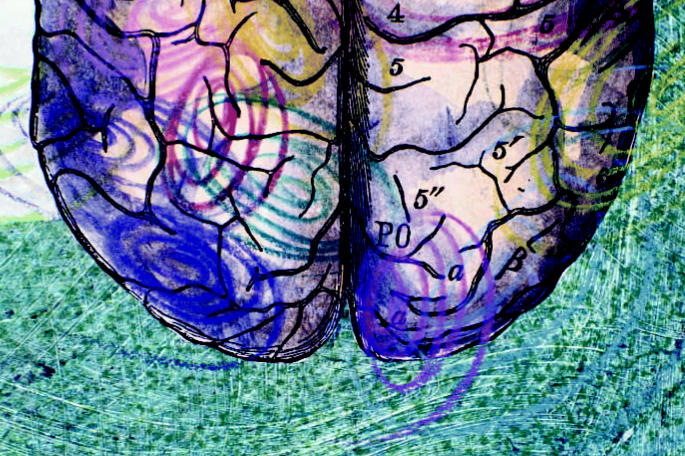
Cognition and cancer. Lead exposure is known to cause declines in brain function, but the link to brain cancers is less clear.

**Figure:**
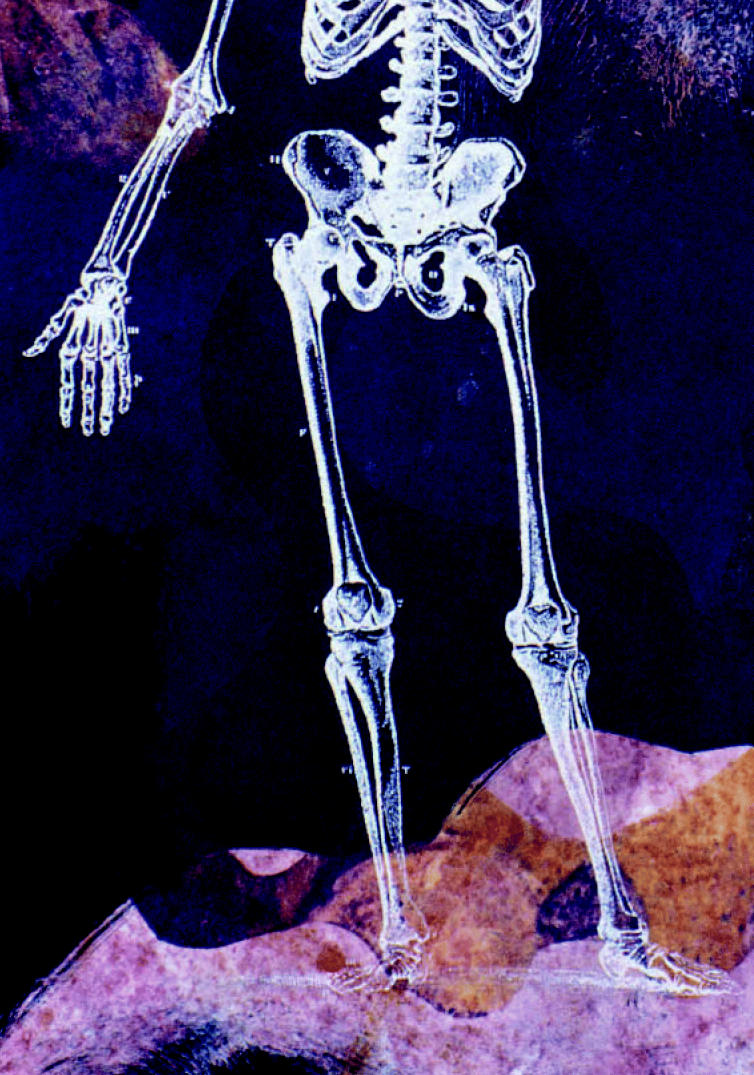
Blood and bone. Measurements of lead in the body are taken from blood and bone, but each has limitations. More accurate methods are needed, particularly to measure effects of low-level exposures.

